# Modern elite winter wheat cultivars use two physiological pathways to achieve yield stability

**DOI:** 10.1093/jxb/eraf191

**Published:** 2025-06-07

**Authors:** Tien-Cheng Wang, Li-Yu Liu, Kirsten Weiß, Tsu-Wei Chen

**Affiliations:** Section of Intensive Plant Food Systems, Albrecht Daniel Thaer-Institute of Agricultural and Horticultural Sciences, Humboldt Universität zu Berlin, Berlin, Germany; Institut für Gartenbauliche Produktionssysteme, Leibniz Universität Hannover, Hannover, Germany; Biometrics Division, Department of Agronomy, National Taiwan University, Taipei, Taiwan; Common Laboratory of Analysis, Albrecht Daniel Thaer-Institute of Agricultural and Horticultural Sciences, Humboldt Universität zu Berlin, Berlin, Germany; Section of Intensive Plant Food Systems, Albrecht Daniel Thaer-Institute of Agricultural and Horticultural Sciences, Humboldt Universität zu Berlin, Berlin, Germany; University of California, Davis, USA

**Keywords:** Agronomic management, field phenotyping, genotypic strategies, physiological mechanism, source–sink balance, stay-green, *Triticum aestivum* L, water-soluble carbohydrate

## Abstract

Identifying target traits for breeding stable, high-yielding winter wheat cultivars is made challenging by the intricate interplay of genotype, environment, and management practices. We hypothesized that yield stability could be achieved through multiple genotypic strategies and that agronomic management stimulating these strategies would enhance stability. To test this, three years of field experiments were conducted using eight high-yielding elite cultivars and three agronomic practices: (i) nitrogen levels (220 or 176 kg N ha^−1^), (ii) fertilizer application timing, and (iii) two sowing dates. Detailed field phenotyping of 130 agronomic, phenological, chemical, and physiological traits resulted in 40 557 measured or derived trait values. Correlation and multivariate analyses suggested that management practices promoting grain number increased yield stability, while nitrogen level influenced the importance of application time and sowing date. Interestingly, modern elite cultivars exhibit two distinct physiological strategies coupling different source capacity and sink demand strategies to achieve genotypic yield stability: (i) coupling high tiller and grain numbers with longer canopy stay-green and higher carbon reserves, and (ii) coupling high grain length with low tiller number and greater remobilization of pre-anthesis carbon reserves. The integration of multiple physiological pathways could therefore facilitate the identification of trait combinations for yield stability breeding.

## Teaser

German winter wheat achieves yield stability by coupling post-anthesis stay-green with high tiller and grain number or coupling high grain length with high re-mobilized pre-anthesis carbon reserves.

## Introduction

Food security is increasingly threatened by climate change and stagnation in yield (Rezaei *et al.*, 2023; Teng *et al.*, 2023). For instance, winter wheat (*Triticum aestivum* L.), which provides about 20% of global protein consumption, has experienced yield stagnation since 2010 with only 0.7% increase per year (Gerber *et al.*, 2024). Selecting wheat cultivars with high and stable performance across diverse environmental conditions is crucial (Macholdt and Honermeier, 2017; Snowdon *et al.*, 2021; Stetkiewicz *et al.*, 2023), yet the physiological mechanisms underlying yield stability remain less understood. The main challenge is due largely to the complex interactions between genotype, environment, and management (G×E×M interactions). Effects of G×E×M interactions on a trait can be quantified by stability indices (SI), which can be static, dynamic, parametric, or non-parametric (Becker and Léon, 1988). However, different types of SI need to be carefully and critically chosen for the research questions (Du *et al.*, 2020; Reckling *et al.*, 2021; Stella *et al.*, 2023) since the correlations between phenotypic traits and yield stability depend on the choice of SI, genotypes, and target population of environments (Wang et al., 2023, 2025). Therefore, one of the challenges for improving yield stability lies in selecting appropriate SIs and representative environments and genotypes.

Breeding stable winter wheat cultivars requires integrated knowledge of target traits that boost yield potential (Fischer, 2011; Beral *et al.*, 2022; Reynolds *et al.*, 2022; Burgess *et al.*, 2023; Fischer *et al.*, 2024), including a comprehensive understanding of source–sink dynamics (Paul *et al.*, 2020; Reynolds *et al.*, 2021, 2022; Weigelt *et al.*, 2021; Murchie *et al.*, 2023; Slafer *et al.*, 2023). Source–sink dynamics governs the balance between the development and growth of different organs (Hilty *et al.*, 2021; Murchie *et al.*, 2023; Slafer *et al.*, 2023) that determine the formation of yield components [grain number (GN) and thousand grain weight (TGW)]. However, different yield components exhibit contrasting sensitivity to source availability (Rosati and Benincasa, 2023). For example, tiller, spike, and GN are highly sensitive to source availability during their critical developmental period (Fischer, 1985; Sabir *et al.*, 2023). In contrast, during the critical period of TGW formation (grain filling), TGW is less responsive to the reduced source availability (Borrás *et al.*, 2004; Serrago *et al.*, 2013; Rivera-Amado *et al.*, 2020). It is still unclear to what extent the traits governing source–sink balance modulate yield stability, and it is likely that multiple physiological mechanisms need to be integrated to maintain a dynamic source–sink balance throughout the critical periods of yield formation to ensure yield stability.

Coordinated source–sink balance during grain development is pivotal (Ma *et al.*, 2023). GN and TGW potentials are considered as the most important components of sink demand after anthesis. Their realization depends on two types of source: post-anthesis photo-assimilates and the remobilization of pre-anthesis carbon reserves, particularly water soluble carbohydrate (WSC) stored in straw (Kühbauch and Thome, 1989; Schnyder, 1993). Under well-watered conditions, post-anthesis photo-assimilates account for more than 60% of grain yield (Masoni *et al.*, 2007; Fang *et al.*, 2021), while pre-anthesis WSC accounts for approximately 20% (Gebbing and Schnyder, 1999). However, pre-anthesis WSC in straw can contribute up to 60% of yield under water deficits that constrain canopy photosynthesis after anthesis (Palta *et al.*, 1994; Yang and Zhang, 2006; Fang *et al.*, 2021). Therefore, it has been reported that pre-anthesis WSC contributes to yield stability under drought (Rebetzke *et al.*, 2008; Slewinski, 2012; Gaur *et al.*, 2022; Kumar *et al.*, 2022). WSC in straw shows complex genetic architecture and low heritability, limiting its utility as a breeding target (Ruuska *et al.*, 2006; Lopes and Reynolds, 2010; Pierre *et al.*, 2010; Ovenden *et al.*, 2017). Most studies focus on WSC concentration at anthesis and its impact for grain weight and yield (Dreccer *et al.*, 2013; Liang *et al.*, 2017; Gaur *et al.*, 2022). However, little attention has been given to the physiological implication of WSC at maturity (Yang *et al.*, 2007; Ovenden *et al.*, 2017). The remaining WSC in straw reflects the integrated net balance between source and sink during post-anthesis and can be an indicator of genotype-specific source–sink dynamics in response to environmental scenarios.

Post-anthesis source capacity is highly related to canopy senescence, which is regulated by genetic control and environmental factors (Thomas, 2013; Distelfeld *et al.*, 2014; Guo *et al.*, 2021). Therefore, breeders select delayed senescence, so-called stay-green traits, to promote yield (Christopher *et al.*, 2016; Chapman *et al.*, 2021; Jocković *et al.*, 2022; Zhou and Yang, 2023), especially under drought conditions (Xie *et al.*, 2016; Kumar *et al.*, 2022). However, in some extreme cases, undesired stay-green—for example, with increased nitrogen fertilizer at heading—can reduce grain filling rate and therefore grain weight and grain yield under well water condition (Yang *et al.*, 2000), resulting in unused WSC in straw at maturity and lower harvest index. This highlights the importance of post-anthesis source–sink balance. Therefore, it can be hypothesized that stable yield can be achieved by coupling physiological characteristics related to source strength and corresponding sink activity at the same time.

The development of source strength and sink demand can be influenced by agronomic managements, such as sowing date and the timing and level of nutrient fertilization. These management strategies offer the potential to optimize the source–sink balance and enhance yield stability (Cooper *et al.*, 2001; Macholdt and Honermeier, 2019; Dueri *et al.*, 2022; Lenoir *et al.*, 2023; Collier *et al.*, 2024; Martre *et al.*, 2024). For example, nitrogen levels affect tiller development (He *et al.*, 2015; Luo *et al.*, 2020), while sowing dates influence phenology and flowering time (Liu *et al.*, 2023), altering the duration of pre- and post-anthesis growth periods (Bogard *et al.*, 2011; Rasmussen and Thorup-Kristensen, 2016; Xi *et al.*, 2023). These factors modify the source–sink dynamics throughout the crop cycle. Thus, it can be hypothesized that management practices that promote source–sink balance could also contribute to achieving yield stability.

In this work, we aim to identify physiological mechanisms of source–sink balance that contribute to yield stability. We conducted three years of field experiments consisting of eight modern elite winter wheat cultivars grown under eight different agronomic management practices. Detailed phenotyping of 130 traits allowed the identification of mechanistic pathways to achieve yield stability and testing of the following hypotheses: (i) stable yield can be achieved by physiological mechanisms coupling source strength with sink demand and (ii) agronomic management practices, by promoting a source–sink balance mechanism, can enhance yield stability.

## Materials and methods

### Plant materials and experimental design

Field trials were conducted for three successive growing years (2018–2019, 2019–2020, and 2020–2021) at the research station of Leibniz University Hannover in Ruthe (52°14′44.3″N, 9°49′1.8″E), Hannover, Germany (Supplementary Fig. S1). Eight elite winter wheat (*Triticum aestivum* L.) cultivars with similar yield level but contrasting yield stability (Wang *et al.*, 2023, 2025) were selected for the study (Supplementary Table S1).

To achieve different growing conditions within one year for the calculation of SI, three agronomic managements (three treatments: nitrogen level, early nitrogen availability, and sowing date) were used in the field experiments, resulting in the field plots arranged in a split–split–split plot design with three replicates (Supplementary Fig. S2A). Each plot had a fixed width of 2 m and contained 15 rows, with a planting density of 330 plants m^−2^. The size of plots in the three consecutive years were 10 m^2^, 14 m^2^, and 14 m^2^, respectively. Border plots surrounding each split–split plot were introduced (Supplementary Fig. S2B) to avoid lateral nitrogen transfer in soil.

The first treatment involved two total nitrogen levels (220 and 176 kg N ha^−1^) representing optimal and sub-optimal nitrogen supply, respectively. The second treatment represented differences in nitrogen availability at the sowing stage. For this treatment, 40 kg N ha^−1^ nitrogen was applied at two time points. In the ‘split’ application, 20 kg N ha^−1^ was applied at sowing and 20 kg N ha^−1^ at heading stage, while in the ‘combined’ application 40 kg N ha^−1^ was applied at the heading stage (Supplementary Table S2). The third treatment used 2–3 week differences in sowing dates (early and late; Supplementary Table S3), creating differences in phenological development and canopy size in early spring. In total, the combinations of three treatments created eight agronomical managements.

Soil nitrogen samples were collected at four evenly selected positions within each replicate with three depths (30, 60, and 90 cm). Fertilizer adjustments were made at the stem elongation stage based on the measured soil nitrogen levels at tillering stage (Supplementary Table S2). To achieve the targeted two total nitrogen levels, we adjusted the amount of fertilizer applied at stem elongation for each treatment (Lichthardt *et al.*, 2020). Plant protection materials were applied according to the standard of intensive wheat production in Germany (Voss-Fels *et al.*, 2019; Sabir *et al.*, 2023).

### Non-destructive measurements and derived parameters

Non-destructive measurements were conducted on a weekly basis after emergence. The following traits were measured: date to developmental stages, leaf area index (LAI), light interception (LI), green canopy area (GCA) and soil plant analysis development (SPAD) value (Supplementary Table S4). The developmental stage of wheat (BBCH9–BBCH87) was investigated using the BBCH scale (Bleiholder *et al.*, 1989; Hack *et al.*, 1992). LAI was measured using a LAI 2200-C plant canopy analyser (LI-COR, Lincoln, NE, USA) with 180° view angle cap and five repeats under the canopy. LI was measured three times below the canopy with an LI-191R Line Quantum Sensor (LI-COR) and one time above canopy with a LI-190R Quantum Sensor (LI-COR). The relative leaf chlorophyll content was estimated after heading from five randomly selected flag leaves with a SPAD-502 meter (Konica-Minolta, Japan).

Green canopy duration (GCD) was determined based on the difference between thermal sum (TT; °Cd) required to reach the heading stage (BBCH61) and TT to reach 50% GCA, which is roughly at the maturing stage (around BBCH79). Longer GCD represents longer stay-green in canopy. The relationship between GCA and TT after anthesis followed a logistic function (Lichthardt *et al.* 2020).


$$\begin{array}{l} TT=\underset{sowing}{\sum }\;\frac{\left(T_{min}+T_{max}\right)}{2}-T_{base} \end{array}$$ (1)

where *T*_min_ and *T*_max_ denote minimum and maximum temperature of a day, and *T*_base_ denotes the base temperature of growth for winter wheat. *T*_base_ is assumed to be 0 in this study.

### Agronomic traits

A dry matter (DM) sample was collected through destructive measurement from a 0.5 m length of a biomass sample cut from a non-boarder row inside each plot and dried in an oven at 70 °C for 2 d. DM samples were collected at six developmental stages: tiller (DM_23_), stem elongation (DM_31_), booting (DM_41_), heading (DM_51_), flowering (DM_61_), and maturity (DM_87_). From the DM_61_ and DM_87_ samples, different plant parts (i.e. straw, spike, and grain) were further separated.

The grain yield was determined by the dry weight of grains from the entire plot harvest sample. Yield components like grain per spike (GpS) and spike number (SN) were investigated from DM_87_ samples. Grain size, including TGW and grain appearance (Supplementary Table S4), was measured with a Marvin seed analyser (GTA Sensorik, Neubrandenburg, Germany).

For nitrogen and WSC analysis, plant biomass samples from the oven were first ground into powder with a laboratory knife mill, Grindomix GM200 (Retsch GmbH, Haan, Germany), at a spin of 10 000 r.p.m. for 1.5 min. An anthrone-based colorimetric method (von Lengerken and Zimmermann, 1991) was used for the determination of WSC, which mainly detects glucose, fructose, disaccharides, and fructosans. The three main steps were as follows: (i) 0.5 g of lyophilized sample powder was added to 100 ml of deionized water and shaken for 1 h; (ii) the sample extract was clarified with a Carrez mixture, filtered, and diluted with mercury (II) chloride solution (0.01%); (iii) anthrone reactant was added to determine WSC concentration using a continuous flow analyser (Scan++, Skalar Analytical, Breda, The Netherlands).

The nitrogen content in powdery samples was determined by elemental analysis using a Vario Max Cube (Elementar Analysensysteme GmbH). The steps involved in the determination of nitrogen content by the combustion method according to Dumas (based on Naumann and Bassler, 1976) were as follows: (i) the sample weight ranged from 50 to 300 mg, depending on the sample material available; (ii) the nitrogen bound in the sample burned under an oxygen supply to molecular nitrogen (N_2_) and a mixture of nitrogen oxides (NO_x_), which was subsequently also reduced to N_2_; (iii) argon was used as the carrier gas, the combustion temperature was 900 °C, and the concentration was determined by means of a thermal conductivity detector.

### Contribution of pre- and post-anthesis reserves to spike dry matter at maturity

The calculation principle described below applies both to DM and total nitrogen (N) content in the spike (Supplementary Table S5). Taking DM as example, the spike DM at maturity (DM_87,spike_) equals the pre-anthesis reserve in the spike (DM_61,spike_) plus the post-anthesis change in the spike (ΔDM_61–87,spike_). ΔDM_61–87,spike_ equals the translocation of DM from the straw (ΔDM_61–87,straw_) plus post-anthesis assimilates (ΔDM_87–61,all_). ΔDM_61–87,straw_ was obtained by the subtraction of DM_87,straw_ from DM_61,straw_. In cases where DM_87,straw_ was larger than DM_61,straw_, the ΔDM_61–87,straw_ was set to zero.

### Environmental data

Environmental data include weather data (i.e. temperature, irradiance, and irrigation), volumetric soil water content (θ_v_), and soil nitrogen content. Weather data were collected hourly throughout the season by the weather station near the study field in the research station (Supplementary Fig. S3). The volumetric soil water content was measured with a Diviner 2000 capacitance sensor (Sentek Pty, Stepney, South Australia) from March to July for all three years. Four diviners were evenly placed along the block centre for each block (replication), resulting in a total of 12 diviners. Weekly measurements were taken at depths ranging from 10 cm to 90 cm below surface, with a 10 cm interval (Supplementary Fig. S4). Soil nitrogen was measured by photometric UV method with the UV-VIS Spectrometer Varian Cary 50 (Varian, USA). In short, soil samples were sieved with mesh and dried in the oven at 100 °C overnight. Sample solution was then diluted to 10 ml with 12.5 mM CaCl_2_. Nitrate and ammonium content were determined at 210 and 270 nm, respectively.

### Statistical analysis

To calculate the yield stability index, the dataset of two years (2020 and 2021) was selected to ensure balanced numbers of genotypes across years for the following analyses. In this study, both a genotypic superiority measure (*P*_i_; Lin and Binns, 1988), which related to high mean performance (lower *P*_i_ values correspond to greater stability), and ecovalence (*W*_i_; Wricke, 1962), which measures total variation, were adopted to describe the stability of traits:


$$\begin{array}{l} P_{i}=\underset{j}{\overset{n}{\sum }}\;\frac{{\left(X_{ij}-M_{j}\right)}^{2}}{2n} \end{array}$$ (2)

$$\begin{array}{l} W_{i}=\underset{j}{\sum }\;{\left(X_{ij}-\bar{X_{i}}-\bar{X_{j}}+\bar{X}\right)}^{2} \end{array}$$ (3)

where *X*_*ij*_ denotes observed trait value of genotype *i* (*i*=1, … , G) in an environment *j* (*j*=1, … , E); *M*_*j*_, the maximum observation in the *j*th environment; ${\bar{X}}_{i}$, the trait mean value for genotype *i*; ${\bar{X}}_{j}$, the trait mean value for environment *j*; and $\bar{X}$, the trait mean value of all observations. The functions *ecovalence()* and *genotypic_superiority_measure()* in ‘*toolStability*’ R-package (Wang and Chen 2023) were used to calculate the two stability indices.

An analysis of variance was performed to test the significance of the treatment effects for each year. A linear model was designated to describe the treatment effects:


$$\begin{array}{l} \begin{array}{lllllllll} Y_{ijklhm}= & \;\mu +\tau_{i}+\beta_{j}+\tau \beta_{ij}+\gamma_{k}\; & +\tau \gamma_{ik}+\beta \gamma_{jk}+\tau \beta \gamma_{ijk}+\delta_{l}\; & +\tau \delta_{il}+\beta \delta_{jl}+\tau \beta \delta_{ijl}+\gamma \delta_{kl}+\tau \gamma \delta_{ikl}\; & +\beta \gamma \;\delta_{jkl}+\tau \beta \gamma \;\delta_{ijkl}+\phi_{h}+\tau \phi_{ih}+\beta \phi_{jh}\; & +\tau \beta \phi_{ijh}+\gamma \phi_{kh}+\tau \gamma \phi_{ikh}+\beta \gamma \;\phi_{jkh}\; & +\tau \beta \gamma \;\phi_{ijkh}+\delta \phi_{lh}+\tau \delta \phi_{ilh}\; & +\beta \delta \;\phi_{jlh}+\tau \beta \delta \;\phi_{ijlh}+\gamma \delta \phi_{klh}\; & +\tau \gamma \delta \phi_{iklh}+\beta \gamma \delta \phi_{jklh}+\tau \beta \gamma \delta \phi_{ijklh}+\varepsilon_{ijklhm}\; \end{array} \end{array}$$ (4)

where *Y*_*ijklhm*_ denotes the value of a trait for cultivar *h* under the agronomical management combination (*jkl*; *j* for total nitrogen level; *k* for application time; *l* for sowing date) from the *i*th block; μ, the mean value of trait observations; τ_*i*_, the random effect of the *i*th block; β_j_, the fixed effect of the *j*th level of nitrogen treatment in the main plot; γ_k_, the fixed effect of the *k*th level of application time in the split-plot; δ_*l*_, the fixed effect of the *l*th level of sowing date in the split–split plot; ϕ_*h*_, the fixed effect of the *h*th level of cultivar in the split–split–split plot; ε, the residuals, which follows a normal distribution with mean 0. Note that the *F*-value of each source of variation was calculated based on its expected mean square (Montgomery, 2017).

Pearson’s correlation coefficient (*r*) was calculated to capture the general correlation of the physiological traits and yield components. In addition, Spearman’s correlation coefficient (Spearman’s *r*) was calculated to compare the ranking of cultivars in stability among traits to avoid the assumption of normality in the ranking. For the phenotypic space analysis, we performed a principal component analysis with scaling.

## Results

### Grain yield between three years and general differences in yield stability between genotypes and managements

We conducted field experiments with eight winter wheat cultivars and eight agronomic management practices over three consecutive years, from 2019 to 2021. Annual mean grain yield across cultivars and management practives was 8.13±0.35 t ha^−1^ in 2019, 7.68±0.63 t ha^−1^ in 2020, and 6.22±0.37 t ha^−1^ in 2021 (Supplementary Table S6). The differences between years can be explained by the characteristics of weather conditions and soil water availability (Supplementary Table S7), which influenced the source–sink balance of plant growth and development at the physiological sub-phases of yield formation. For instance, plants in 2021, a dry year, reached maturity approximately 130 °Cd earlier than in 2020 (Supplementary Table S6), due to an ~−30 °Cd shorter vegetative phase (sowing–BBCH31), an ~−95 °Cd pre-anthesis reproductive phase (BBCH31–BBCH61), and similar post-anthesis phase (BBCH61–BBCH87). Also, uneven distribution of precipitation was observed in 2021. Between tillering and stem elongating, soil water availability in the top 30 cm (θ_v_30_) was only 9% in 2021, which was 42% lower than the other two years (Supplementary Fig. S4A), resulting in a reduced DM at booting stage (2020: 7.9±1.5 t ha^−1^; 2021: 6.4±0.92 t ha^−1^; Supplementary Table S6) but increased tiller number (2020: 5.1±0.91×10^6^ ha^−1^; 2021: 7.4±1.5×10^6^ ha^−1^, Supplementary Table S6). In additions, there was nearly no rain (0.13 mm) during the flowering period (BBCH61–BBCH71) and 100 mm during canopy senescence (BBCH71–BBCH87).

**Fig. 1.**
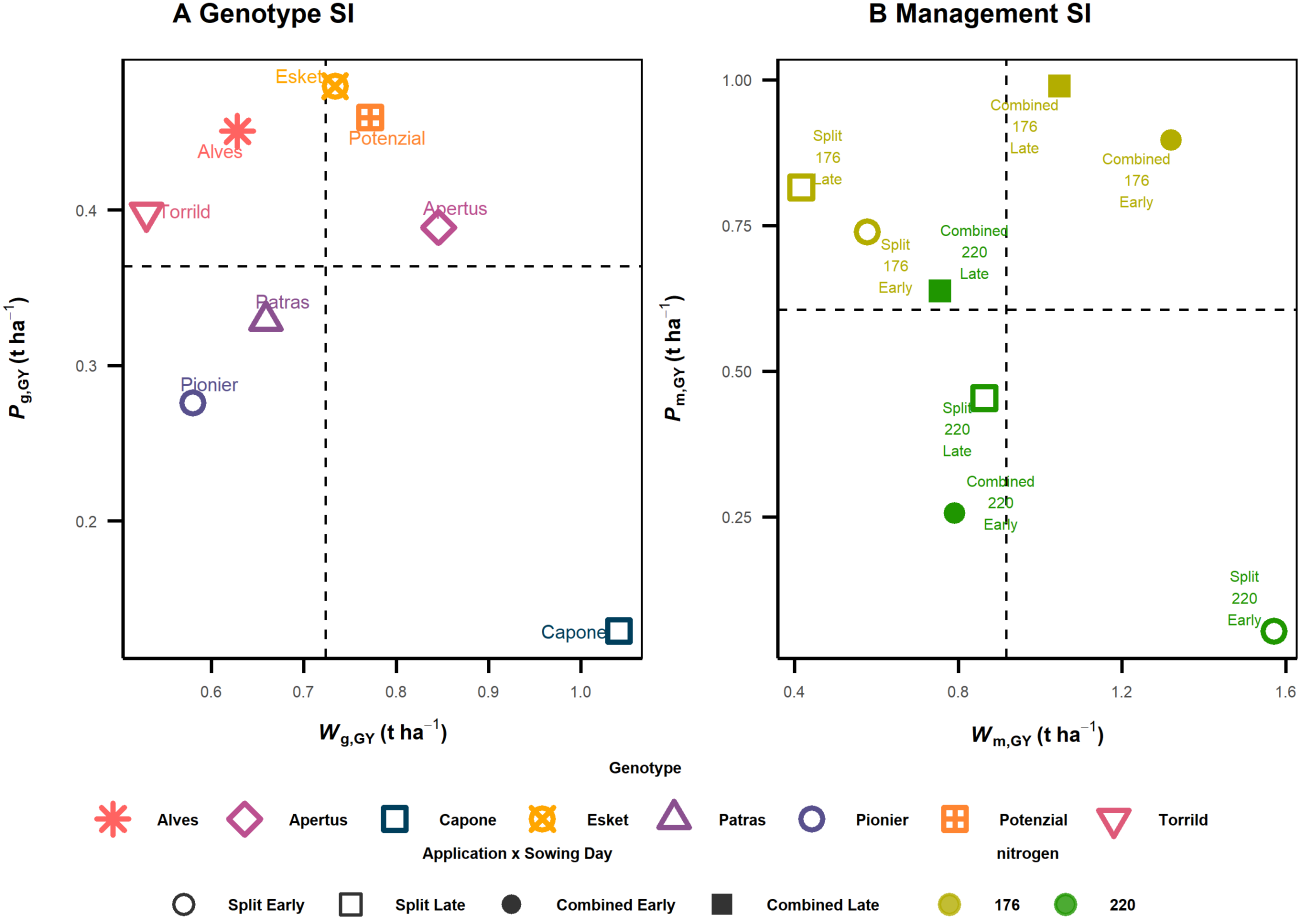
Scatter plot of two yield stability indices for genotype and management perspectives from field experiments from 2020 to 2021. Two kinds of stability indices are a genotypic superiority measure (*P*_i_) and ecovalence (*W*_i_). Note that lower *P*_i_ values correspond to greater stability. Stability indices of grain yield were calculated based on two perspectives: genotype (A; *P*_g_ and *W*_g_) and management (B; *P*_m_ and *W*_m_). The lower the stability index value, the more stable the cultivar or management. Colours and shapes in (A) refer to eight cultivars. Open and filled symbols in (B) signify split and combined application of heading fertilizer, respectively. Circles and squares in (B) signify early and late sowing dates, respectively. Yellow-green and green colours signify sub-optimal (176 kg ha^−1^) and optimal (220 kg ha^−1^) total nitrogen, respectively, in (B). Dashed lines represent the mean value of each axis.

The effect of G×E×M interactions on yield stability was studied from the perspectives of genotype and management. For this, only the data from 2020 and 2021 were used to ensure the balanced numbers of cultivars and managements. Two contrasting SIs, superiority index (*P*_i_) and ecovalence (*W*_i_), were chosen in this study to represent the relevance of mean performance and variance, respectively. Note that *P*_i_ is highly correlated to mean trait performance, and lower *P*_i_ values correspond to greater stability, which measures the performance and stability simultaneously. In contrast, *W*_i_ has no correlation to mean trait performance, which measures the variation only. For the genotypic superiority index for grain yield (*P*_g,GY_), Capone and Esket were identified as the most (lowest *P*_g,GY_) and the least yield stable (highest *P*_g,GY_) cultivars, respectively (Fig. 1A). The management superiority index (*P*_m,GY_) was mainly determined by the total nitrogen levels—optimal nitrogen level was more stable (lower management superiority index; Fig. 1B). It is noteworthy that interactions were observed between agronomical management practices on *P*_m,GY_. For instance, early sowing date contributed more to management stability than split application time under optimal nitrogen level, while it was the other way around under the sub-optimal nitrogen level (Fig. 1B). In general, management combining early sowing date and split nitrogen application demonstrated the highest stability for both total nitrogen levels, which can be recommended for yield stable management.

### Stability in thousand grain weight explained the most genotypic variation in yield stability, while the management that promotes grain number increased yield stability.

**Fig. 2.**
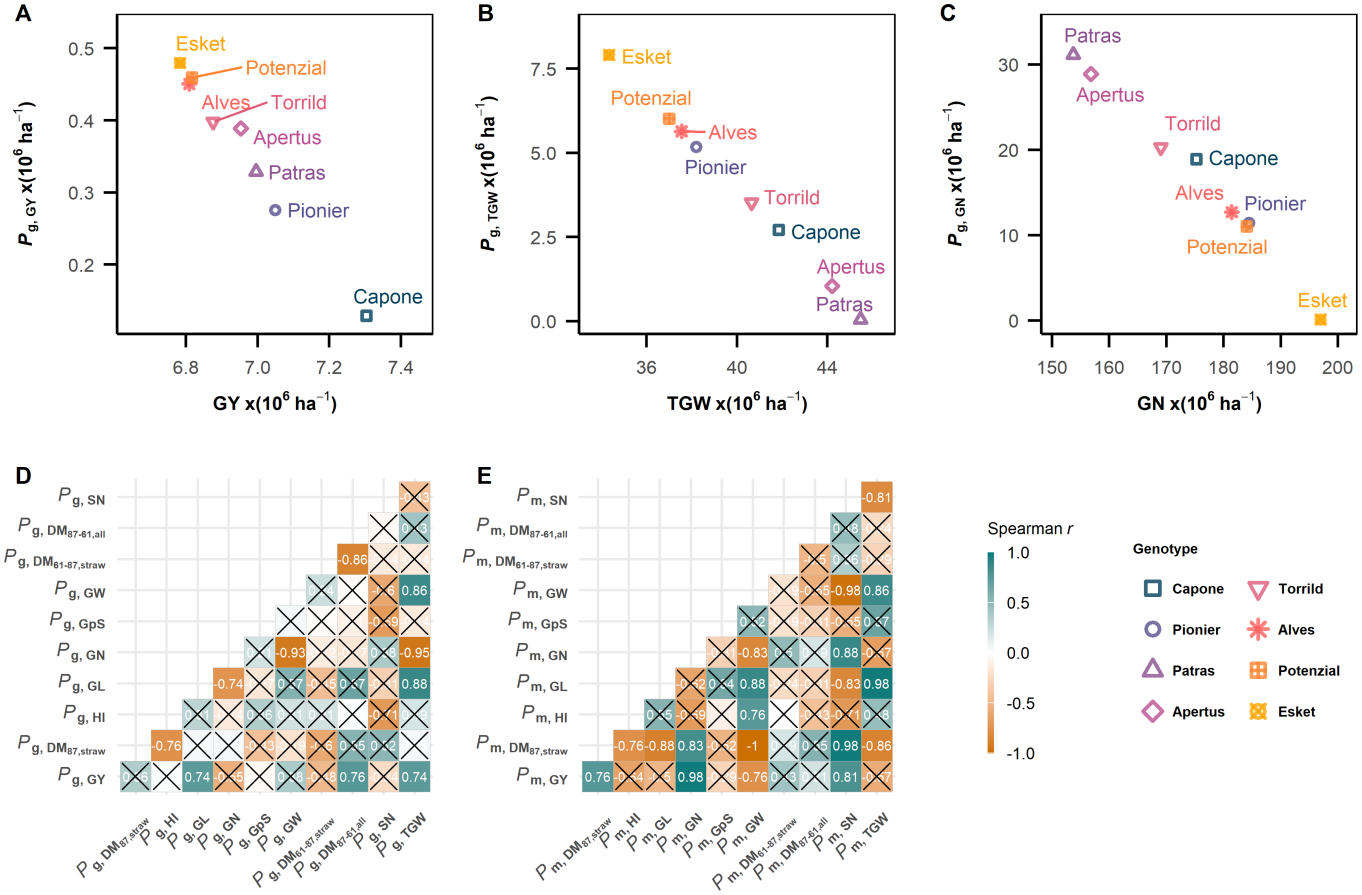
Ranking of superiority index (*P*_i_) in yield and eight agronomic traits. (A–C) Scatter plot of genotypic stability index (*P*_g_) versus mean trait in grain yield (GY; A), thousand grain weight (TGW; B), and grain number (GN; C). (D, E) Spearman’s correlation matrix among nine traits in genotypic superiority index *P*_g_ (D) and in management superiority index *P*_m_ (E). Coloured squares with white text represent significant Spearman correlations (*P*<0.05), viridian green signifies a positive correlation and brown signifies a negative correlation. Non-significant correlations are marked with a black cross. Traits are abbreviated as follows: ΔDM_61–87,straw_, remobilized DM from straw; ΔDM_87–61,all_, post-anthesis assimilates; DM_87,straw_, straw dry matter at maturity; GL, grain length; GN, grain number; GpS, grain per spike; GW, grain width; GY, grain yield; HI, harvest index; SN, spike number; TGW, thousand grain weight.

The superiority index of a trait (*P*_i_) is well-correlated with the mean of the traits, representing stability and trait performance simultaneously. This was shown in grain yield (GY; Fig. 2A), TGW (Fig. 2B), and GN (Fig. 2C). Interestingly, the most stable cultivars, Capone, Pionier, and Patras ranked differently in their superiority index of TGW (*P*_g,TGW_) and GN (*P*_g,GN_), indicating potential differences in genotypic strategies of yield formation to achieve yield stability. In general, cultivars showed higher stability in TGW had a lower stability in GN (Fig. 2B, C). For instance, Patras, ranked first in *P*_g,TGW_ and eighth in *P*_g,GN_. By contrast, the least stable cultivar, Esket, ranked first in *P*_g,GN_ and eighth in *P*_g,TGW_.

To investigate the relationships among *P*_g_ of agronomic traits, Spearman’s correlation was used to avoid the assumption of a normal distribution in *P*_g_. Our results revealed that *P*_g,GY_ was positively correlated with *P*_g,TGW_ (Spearman’s *r*=0.74) instead of *P*_g,GN_ (Spearman’s *r*=−0.35). *P*_g,TGW_ was positively correlated with *P*_g_ of its component grain width (*P*_g,GW_; Spearman’s *r*=0.86) and grain length (*P*_g,GL_; Spearman’s *r*=0.88). For the relationships between GY and the components of TGW, it was unexpected that GY correlated to grain length (GL; Spearman *r*=0.74) as strongly as TGW, while no relation between GY and GW was found. Interestingly, *P*_g,GN_ was not explained by the *P*_g_ in its components (*P*_g,__sn_ and *P*_g,GpS_) and neither *P*_g,__sn_ nor *P*_g,GpS_ explained the *P*_g,GY_ (Spearman’s *r*=−0.24 and −0.05, respectively). These results indicated that yield stability in the studied cultivars is more likely to be achieved by a stable TGW, which is realized by the stability in GL.

Different from *P*_g,GY_, the management superiority index of GY (*P*_m,GY_) was primarily explained by *P*_m,GN_ (Spearman’s *r*=0.98; Fig. 2E), while there was no significant correlation between *P*_m,GY_ and in *P*_m,TGW_ (Spearman’s *r*=−0.57). This suggested that management that promotes the development of GN results in yield stability. This is in accordance with the observation that stability of SN (*P*_m,SN_) and straw dry matter at maturity (*P*_m,DM87,straw_) were both positively correlated with *P*_m,GY_ (Spearman’s *r*=0.81 and 0.76, respectively). Interestingly, stable GN for a management was better explained by stable straw and SN, not by GpS. That is, the management that promotes tiller growth in turn promoted SN and GN, and ultimately GY. This also explains the interactions between agronomical managements on *P*_m,GY_ (Fig. 1B), since GN was increased by early sowing date under optimal nitrogen level and by split application time under sub-optimal nitrogen.

### Variations in yield stability among cultivars are primarily due to differences in their post-anthesis assimilates

**Fig. 3.**
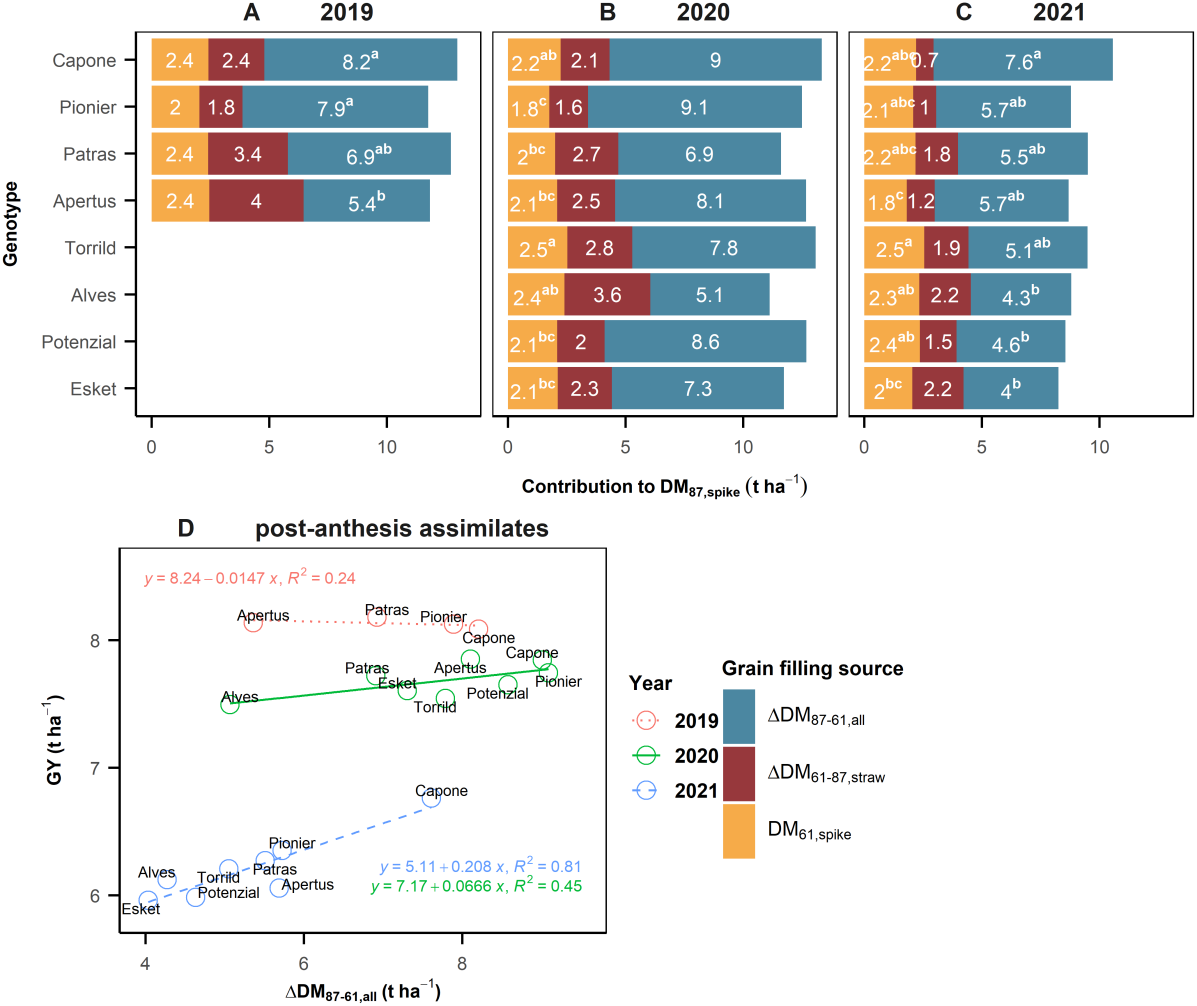
Contribution of grain filling sources to spike dry matter at maturity from 2020 to 2021. (A, B) Dry matter in spike at maturity (DM_87,spike_) in 2020 (A) and 2021 (B). Coloured filled bars represent three contributing sources in DM_87,spike_: spike dry matter at anthesis (DM_61,spike_; orange), remobilized DM from straw (ΔDM_61–87,straw_; blue), and post-anthesis assimilates (ΔDM_87–61,all_; yellow). Different letters denote significant difference at *P*=0.05 across cultivars from split–split–split plot ANOVA analysis. (C) correlation of individual years of ΔDM_87–61,all_ to grain yield. The sorting of the cultivars is based on their genotypic superiority index of grain yield (*P*_g,GY_).

The realization of spike weight at maturity (DM_87,spike_) required supply from three sources: spike dry matter at anthesis (DM_61,spike_), remobilized dry matter (ΔDM_61–87,straw_) and post-anthesis assimilates (ΔDM_87–61,all_). The contributions of these three sources can be quantified by comparing the dry weight in spike and in straw at anthesis and maturity. Overall, ΔDM_87–61,all_ contributed 59% of DM_87,spike_ (average across years, cultivars and managements), while the contributions from ΔDM_61–87,straw_ (19.6%) and DM_61,spike_ (21.3%) were similar. Analysis of variance suggested that ΔDM_87–61,all_ only differed between cultivars in the dry year 2021, and the most stable cultivar, Capone, had 3.3–3.6 t ha^−1^ more post-anthesis assimilates than the least stable cultivars, Alves, Potenzial, and Esket (Fig. 3A, B). Despite the differences in pre-anthesis weathering conditions between 2020 and 2021, the absolute values in DM_61,spike_ of each cultivar showed only subtle variation, while the major reduction of GY in 2021 was mainly caused by the reduction in remobilization and post-anthesis photo-assimilates. In the three sources of spike weight, only ΔDM_87–61,all_ showed positive correlations with GY in 2020 and 2021 (Fig. 3C), confirming the vital contribution of post-anthesis assimilates to yield.

### Stay-green increases post-anthesis assimilates and stimulates cultivar-specific strategies of carbohydrate dynamics

**Fig. 4.**
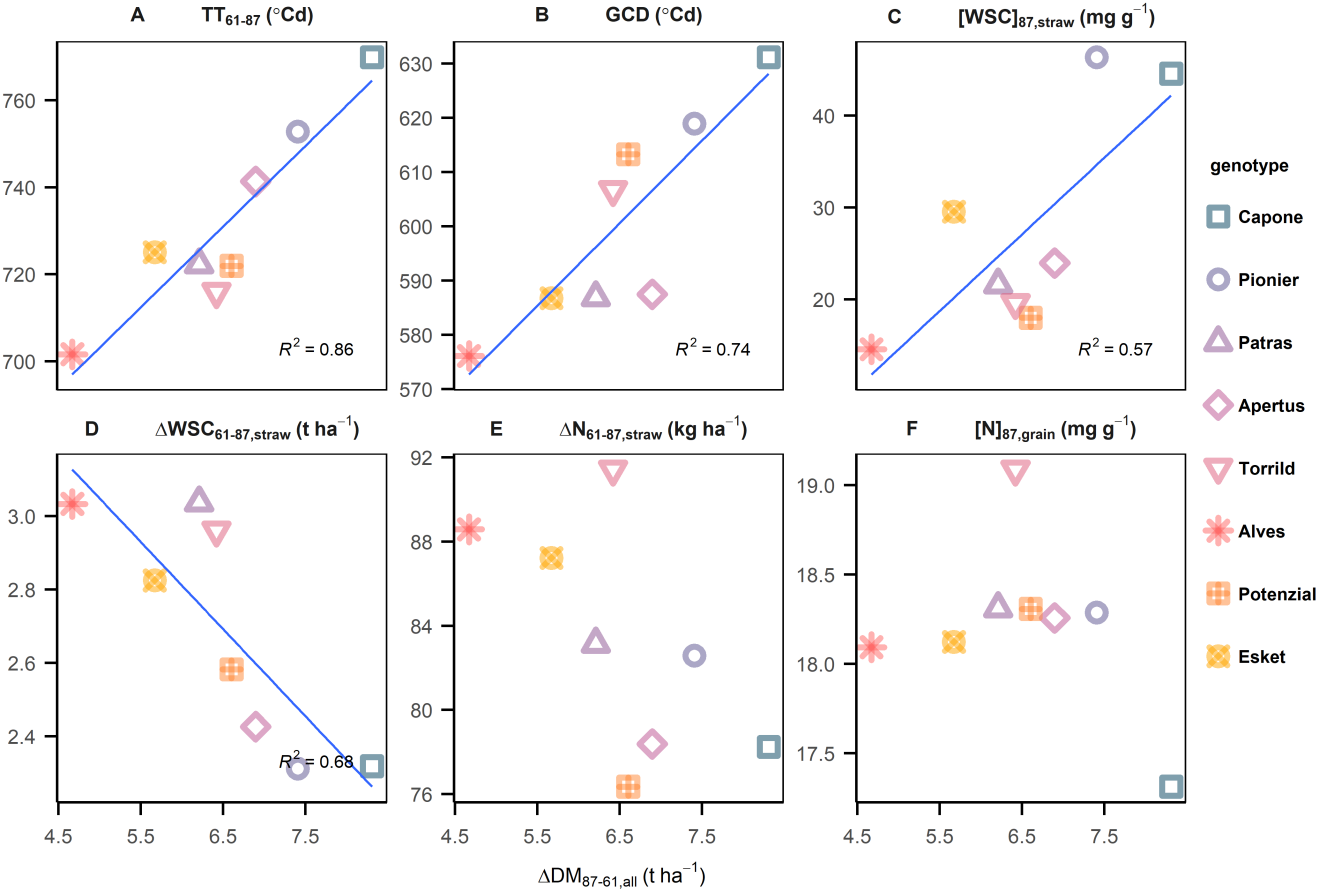
Relationships between post-anthesis assimilates and six physiological trait averages between 2020 and 2021. Positive correlations (A–C) and negative correlations (D–F). Abbreviation of trait names: ΔDM_87–61,all_, post-anthesis assimilates; ΔN_61–87,straw_, remobilized nitrogen from straw; ΔWSC_61–87,straw_, remobilized water soluble carbohydrate from straw; DM_87,straw_, dry matter in straw at maturity; GCD, green canopy duration; [N]_87,grain_, nitrogen concentration in grain at maturity; TT_61–87_, thermal sum between anthesis and maturity; [WSC]_87,straw_, water soluble carbohydrate concentration in straw at maturity. Blue line denotes the regression line when the *P*-value of regression is significant at level α=0.05.

**Fig. 5.**
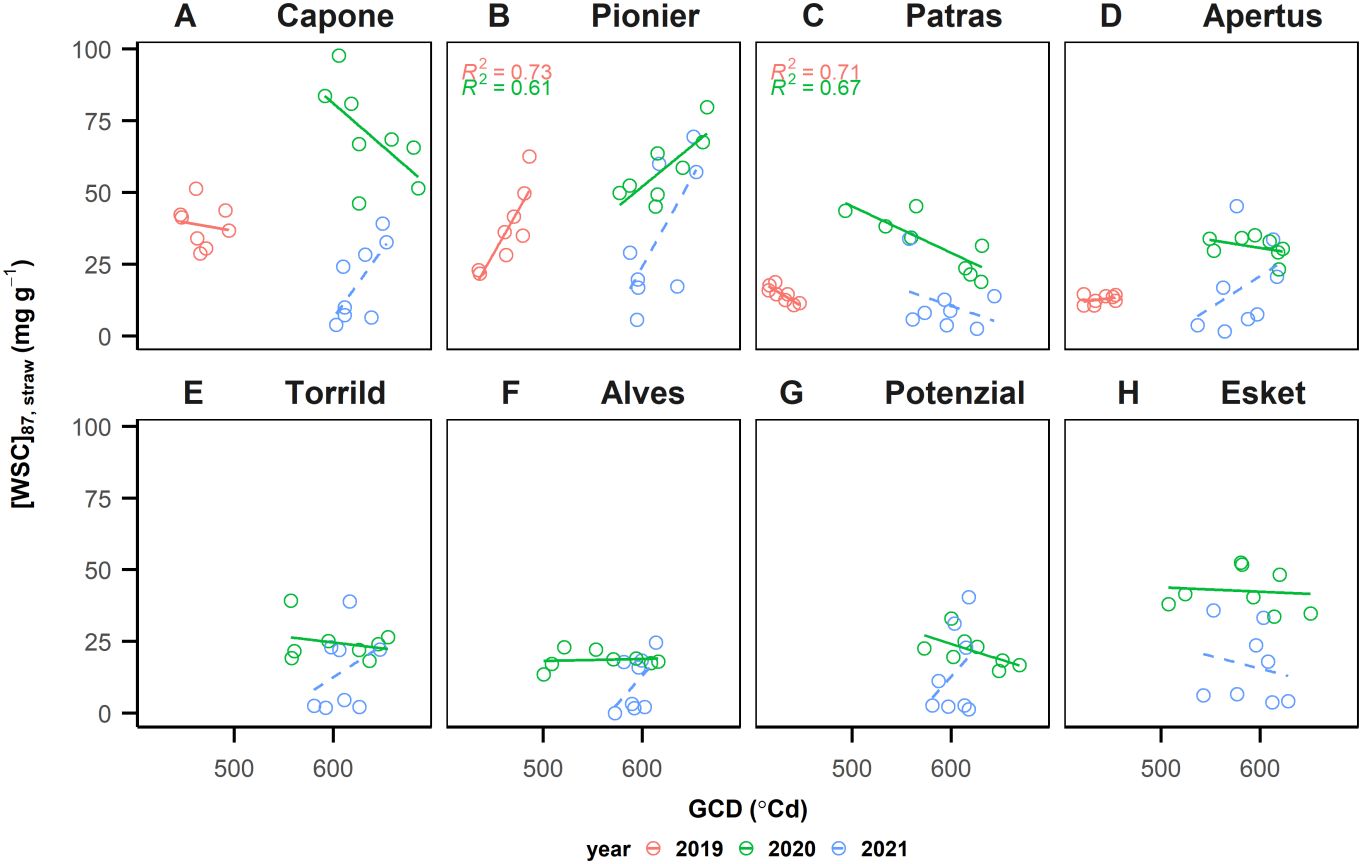
Genotypic-specific relationships of balance between post-anthesis source (green canopy duration, GCD) and sink (water soluble carbohydrate concentration in straw at maturity, [WSC]_87,straw_) in 2020 and 2021. (A–H) Eight genotypes sorted with yield stability (genotypic superiority measure; *P*_g_). Each point represents the mean of three samples (*n*=3). Green colour and solid line for 2020 and blue colour and dashed line for 2021. Note that only the regressions with significant slope (*P*<0.05) are shown with *R*^2^ values. Each point represents the mean value of a management.

Because of the importance of ΔDM_87–61,all_, its correlation with other post-anthesis physiological traits was examined. Across years and managements, ΔDM_87–61,all_ correlated positively with post-anthesis phase thermal sum (TT_61–87_, *R*^2^=0.86; Fig. 4A) and green canopy duration (*R*^2^=0.74; Fig. 4B), which means the stay-green period and post-anthesis growing time stimulate post-anthesis assimilates. Surprisingly, ΔDM_87–61,all_ positively correlated with the WSC concentration in straw at maturity ([WSC]_87,straw_, *R*^2^=0.57; Fig. 4C) and negatively correlated with the remobilized WSC in straw (ΔWSC_61–87,straw_, *R*^2^=0.68; Fig. 4D). This suggests that, in stable elite cultivars, (i) post-anthesis assimilates are the preferred source for grain filling, (ii) post-anthesis source capacity exceeds sink demand and is therefore not fully utilized, or (iii) straw may serve as a potential sink organ after anthesis. In contrast to WSC, ΔDM_87–61,all_ correlated neither with re-mobilized nitrogen from straw (ΔN_61–87,straw_; Fig. 4E) nor nitrogen concentration in grain ([N]_87,grain_; Fig. 4F). We further examined the differences of post-anthesis carbohydrate dynamics between cultivars, comparing GCD with WSC concentration in straw at maturity ([WSC]_87,straw_). The GCD is calculated from the difference between the time of anthesis in terms of thermal sum and the time of 50% canopy senescence, which is around 2086–2336 °Cd, close to BBCH79 and BBCH86. Notably, the most stable three cultivars, Capone, Pionier, and Patras, showed the larger variations in [WSC]_87,straw_ within a year and higher responsiveness of [WSC]_87,straw_ to GCD than the less stable cultivars (Fig. 5). Assuming that GCD represents the source strength at post-anthesis (Fig. 4B), a cultivar with a positive slope of [WSC]_87,straw_ to GCD experiences an oversupply of assimilates when management practices extend its GCD. In contrast, negative slope indicates that sink demand exceeds the source provided by current photosynthesis, leading to remobilization. Interestingly, the three most stable cultivars exhibited different slopes (Fig. 5A–C). The most stable genotype, Capone, displayed flexibility in slope between 2020 and 2021 (Fig. 5A). The other two stable cultivars, Pionier and Patras, showed contrasting slopes (Fig. 5B, C), suggesting different physiological strategies in post-anthesis carbohydrate dynamics.

### Two genotypic strategies involve contrasting source–sink balance for achieving yield stability

**Fig. 6.**
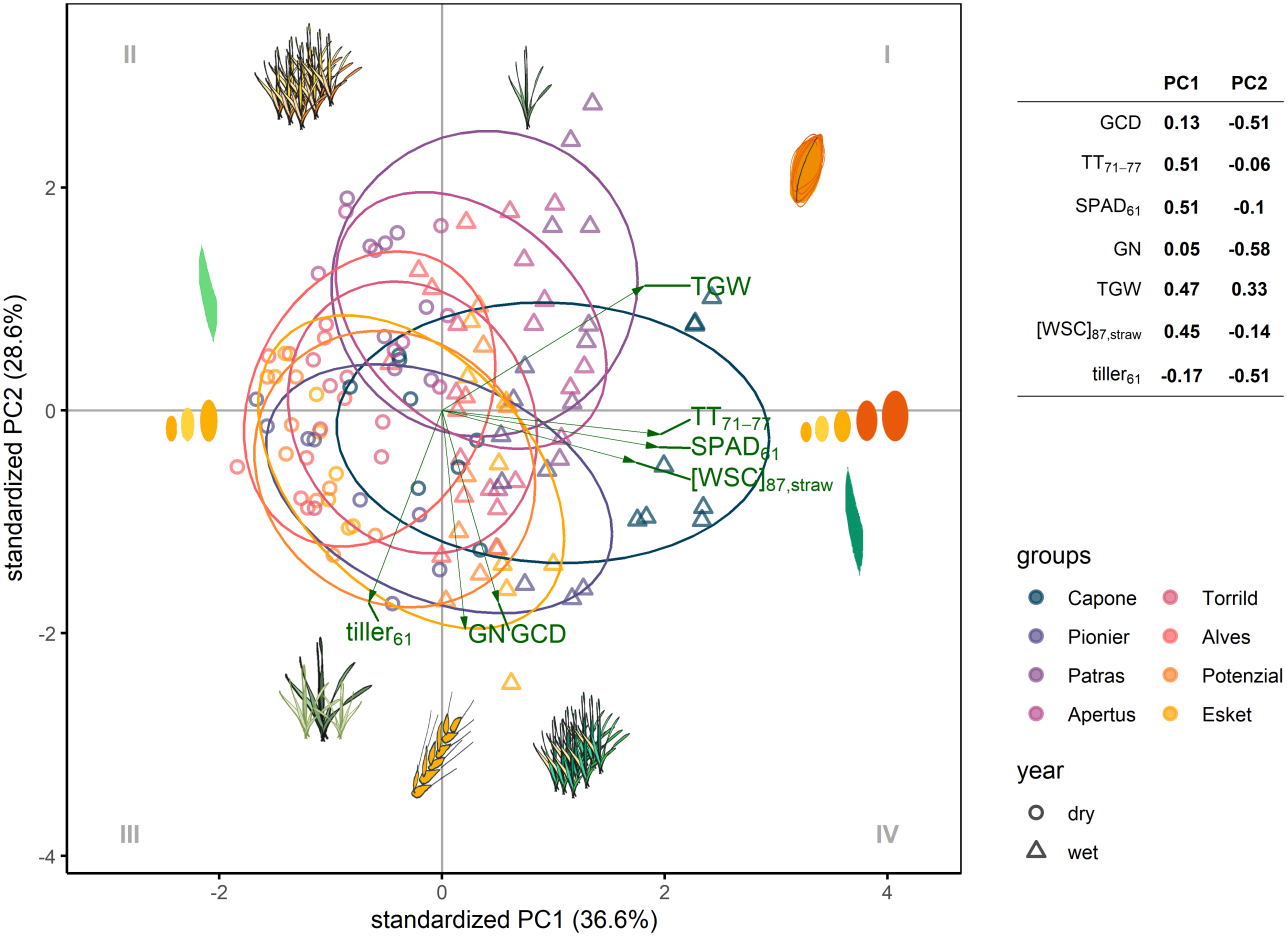
The projection of eight cultivars on phenotypic space defined by first two principal components (PC) from 2020 to 2021. Seven traits are the following: GCD, the thermal sum of green canopy duration (°Cd); SPAD_61_, the chlorophyll content in flag leaf at anthesis; TT_71–77_, the thermal sum of grain filling duration (°Cd); GN, grain number (×10^6^ ha^−1^); tiller_61_, tiller number at anthesis; TGW, thousand grain weight (g/1000 grain); [WSC]_87,straw_, water soluble carbohydrate concentration in straw at maturity (mg g^−1^). Open triangle and circle refer to wet (2020) and dry (2021), respectively. Grey Roman numerals represent the quadrants.

To explore differences in physiological mechanisms between cultivars and managements to achieve yield stability, a post-anthesis phenotypic space was analysed based on principal components analysis, comprising seven source–sink traits related to GY. The first two principal components (PC) explained 65.2% of the variability of phenotypic space (Fig. 6). PC1 (36.6%) correlated positively with the grain filling duration (TT_71–77_), leaf chlorophyll content at anthesis (SPAD_61_), TGW, and WSC concentration in straw at maturity ([WSC]_87,straw_). PC1 represented a gradient from cultivars with stronger source capacity—supporting longer grain filling duration and more carbon deposition in sink organs (grain and straw, right hand side of PC1)—to cultivars with weaker source capacity—relying on shorter grain filling duration and more carbon remobilization from straw (left hand side of PC1). PC1 distinguished the most stable cultivar, Capone, from the others, while also separating the different year conditions (Fig. 6). PC2 explained 28.6% of the variability of phenotypic space and was negatively correlated with GCD, tiller number at anthesis (tiller_61_) and GN, but positively correlated with TGW (Fig. 6). PC2 represented a gradient from cultivars with lower tiller, lower GN, and shorter GCD—relying on WSC remobilization to maintain TGW (upper parts of PC2)—to cultivars with more branching, longer GCD, and using more post-anthesis assimilates to maintain high GN (lower parts of PC2). PC2 differentiated the genotypic stability between the third and eighth ranked cultivars. Overall, yield stability was primarily secured by cultivars that realized sink capacity TGW through carbon translocation (diagonal direction from quadrant III to I), while stay-green cultivars achieve stability by reducing grain abortion rate with higher source capacity at anthesis and thereafter, indicating by the accumulation of [WSC]_87,straw_ (quadrant IV). For the principal component analysis, source traits related to light harvesting—like LAI and light interception—were excluded since they made minimal improvement to the variance explained, probably due to the fact that LAI and LI in the elite cultivars have been already optimized (Lichthardt *et al.*, 2020).

**Fig. 7.**
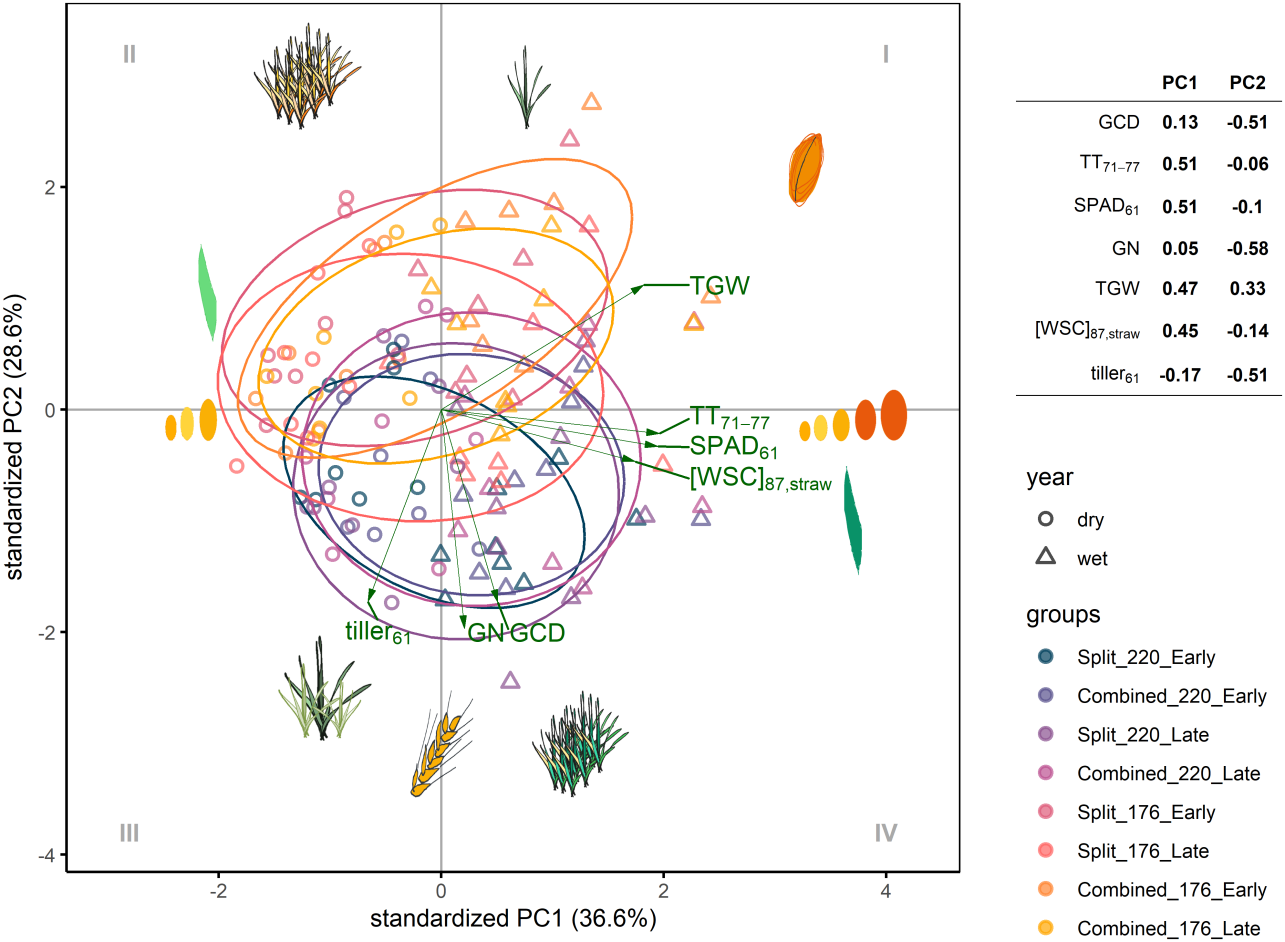
The projection of eight managements on phenotypic space defined by first two principal components (PC) from 2020 to 2021. The seven traits are the following: GCD, thermal sum of green canopy duration (°Cd); SPAD_61_, chlorophyll content in flag leaf at anthesis; TT_71–77_, thermal sum of grain filling duration (°Cd); GN, grain number (×10^6^ ha^−1^); tiller_61,_ tiller number at anthesis; TGW, thousand grain weight (g/1000 grain); [WSC]_87,straw_, water soluble carbohydrate concentration in straw at maturity (mg g^−1^). Open triangle and circle refer to wet (2020) and dry (2021), respectively. Grey Roman numerals represent the quadrants.

In contrast, PC1 did not differentiate the phenotypic spaces between managements, and the differences between managements were predominantly explained by PC2 (Fig. 7). This is in accordance with the observation that managements enhancing GN—through increased tiller number, SN, and extended CGD—improved management stability (Fig. 2).

## Discussion

In this study, we investigated yield stability in winter wheat through a detailed phenotypic analysis of 130 traits observed in three years of field experiments with eight different agronomic management practices. This comprehensive approach enabled us to examine the influence of both genotypic and management factors on yield stability (Fig. 1), focusing on a selection of eight modern elite cultivars with comparable high yield levels (Voss-Fels *et al.* 2019). Our analysis of these high-performing cultivars, distinct from comparing a panel of breeding progress, reveals three key insights into the mechanisms underlying yield stability in modern winter wheat: (i) stability ofTGW explained the largest portion of genotypic variation in yield stability (Fig. 2D); (ii) management strategies promoting GN are effective in enhancing yield stability (Fig. 2E); and (iii) different physiological strategies among modern cultivars were identified, each physiological strategy leveraging a unique source–sink balance to achieve stable yields (Fig. 6).

### Modern cultivars exhibit two distinct physiological pathways coupling different source and sink capacity to achieve genotypic yield stability

Understanding the physiological basis of yield components is essential for studying yield stability (Fischer, 2011). Based on the sink-limitation theory during post-anthesis, conventional breeding has focused on increasing GN as a target trait (Fischer, 2008; Sreenivasulu and Schnurbusch, 2012; Slafer *et al.*, 2022). Despite similar genotypic variation in GN and TGW between the cultivars used in this study (27% and 29%, respectively; Fig. 2), our results showed that stable GL leads to stable TGW, which contributes more to genotypic yield stability than GN in modern cultivars (Fig. 2B, D). Since TGW is highly heritable (Sadras and Slafer, 2012), high TGW could be an indicator to distinguish yield stability across modern cultivars. The high heritability of TGW likely stems from the stability of GL (Brinton *et al.*, 2017; Calderini *et al.*, 2021), as variations in GL across managements and years are much smaller than those observed for grain width (Supplementary Fig. S5). However, Capone and Pionier, the cultivars with the most stable yields, exhibit neither the highest TGW nor the highest GN. This suggests that achieving yield stability may require multiple trait combinations instead of a single trait.

The phenotypic space among cultivars indicates two potential trait combinations for achieving yield stability (Fig. 6): (i) coupling high tiller number and GN with longer canopy stay-green, leading to high water soluble carbohydrate in stem at maturity ([WSC]_87,straw_) (Capone and Pionier, designated as physiological strategy 1, PS1), or (ii) coupling high GL with low tiller number and enhanced remobilization of pre-anthesis carbon reserves and less [WSC]_87,straw_ (Patras, designated as physiological strategy 2, PS2). PS1 and PS2 represent distinct strategies of canopy development and grain filling, highlighting the diverse opportunities to confer yield stability.

In PS1, high post-anthesis assimilates are mainly determined by the longer GCD (Fig. 4), suggesting a strong post-anthesis source strength (Parry *et al.*, 2011; Lichthardt *et al.*, 2020; Jaenisch *et al.*, 2022). This has been matched with the recent finding that breeding progress in stay-green traits improves yield by sustaining GN (Chapman *et al.* 2021), probably through better root development and enhanced nitrogen uptake and use efficiency, thereby increasing tiller survival and effective SN (Ding *et al.*, 2023). In PS2, high GL in Patras could be linked with its large carpel size (Brinton and Uauy, 2019). Since Patras showed reduced tiller number and reduced SN (Fig. 6) but high pre-anthesis WSC in straw ([WSC]_61,straw_), it might develop more cells or larger cell size in the canopy for carbon storage (Pierre *et al.*, 2010), finally leading to high TGW (Dreccer *et al.*, 2009; Liang *et al.*, 2017). This indicates a potential link between storing pre-anthesis WSC and development of large carpels. The remobilized WSC in Patras (Fig. 4D) compensates its short GCD and weak post-anthesis source strength (Fig. 5C), thereby maintaining yield level (Fig. 3B, C) and yield stability (Figs 1, 2).

It is interesting that the second least stable cultivar, Potenzial, exhibited similar tiller number, GpS, GN (sink strength), and GCD (post-anthesis source strength) to the second stable cultivar, Pionier. However, Potenzial had a smaller grain area, lower TGW, and lower GY than Pionier (Supplementary Fig. S5). A key difference was in the total duration of their post-anthesis period (BBCH61–BBCH87): Potenzial had a shorter post-anthesis duration, similar to Patras, while Pionier had significantly longer post-anthesis duration (Supplementary Fig. S6). The similar GCD of Pionier and Potenzial highlights the importance of canopy duration after GCD for realizing yield potential in modern cultivars. When both BBCH61–GCD and GCD–BBCH87 are long (>~750 °Cd), stay-green enhances yield stability, as observed in Capone and Pionier, as reflected in higher WSC accumulation in straw ([WSC]_87,straw_). In contrast, when BBCH61–BBCH87 is relatively short (<~720 °Cd), cultivars such as Patras benefit more from remobilizing pre-anthesis reserves to compensate for assimilate loss caused by senescence. However, if BBCH61–GCD is long while GCD–BBCH87 is short, as in Potenzial, yield potential remains underutilized due to limited time for translocation, restricting grain filling and grain weight. These finding emphasize the importance of trait combinations that integrate phenology, stay-green, carbon dynamics, and yield components, providing a clearer framework to distinguish physiological strategies across modern cultivars. Considering combinations of traits and environmental conditions together provides the potential to reconcile the controversies regarding the effect of grain filling source on yield and yield stability (Murchie *et al.*, 2023). For instance, there is no consensus on relationships between stay-green traits and yield gain due to the stay-green dependence on environmental conditions, multiple physiological pathways, and the varying definitions of stay-green (Chapman *et al.* 2021; Antonietta *et al.*, 2024).

In the studied cultivars, contrasting physiological strategies might also suggest differences in water use capacity. PS1, which had higher yield stability, more tillers, and a longer GCD, may rely on greater water retention during the post-anthesis period (Fang *et al.*, 2021). This aligns with its significantly longer flowering and post-anthesis phases compared with PS2 (Supplementary Fig. S6). It seems that PS1 does not follow the traditional recommendation for drought avoidance of early flowering (Shavrukov *et al.*, 2017; Verrico and Preston, 2025). However, we would like to emphasize that the importance of drought avoidance depends on the environmental scenario (Tardieu, 2012) and the conditions in 2021 were not extremely dry. In contrast, PS2, with fewer tillers and earlier flowering, may achieve yield stability by reducing the transpiration requirement at canopy level, while maintaining photosynthesis at leaf level to ensure pre-anthesis carbon reserves (Borrell *et al.*, 2014, 2023). This is in accordance with our unpublished data showing that Patra has low stomatal density but larger stomatal size. These finding suggest that the two physiological strategies might be coupled with different water-use strategies for achieving yield stability, offering valuable insights for drought-resistance breeding (Vadez *et al.*, 2024).

Future crop breeding for yield stability could benefit from selecting candidates with trait combinations identified in PS1 and PS2, which integrate post-anthesis phase, stay-green traits, canopy development, and carbohydrate dynamics. A recent study (Pao *et al.*, 2023) demonstrated that optimal trait combinations enhance canopy productivity. Specifically, it highlighted the importance of coordinated canopy photosynthetic acclimation and canopy architecture, with selection patterns varying depending on breeding environments. Exploring the genetic architecture (Cao *et al.*, 2020) and identifying alleles underlying these strategies could facilitate molecular marker-assisted breeding and genomic selection (Voss-Fels *et al.*, 2019). A multi-allelic trait introgression or hybrid approach could be used to develop progeny with improved yield stability and optimized source–sink balance (Longin and Reif, 2014; Han *et al.*, 2021). Furthermore, to better evaluate physiological strategies for yield stability and distinguish cultivar-specific responses, future studies should consider multiple physiological pathways that influence reproduction under environmental fluctuations (Pagnussat and Gomez-Casati, 2024). Key physiological traits for further investigation include tiller morphology (Dreccer *et al.* 2013; Manntschke *et al.* 2025), tiller fertility (Ding *et al.* 2023), spike photosynthesis (Molero and Reynolds, 2020), remobilization rate (Xie *et al.*, 2016), enzyme activity related to starch synthase (Borrill *et al.*, 2015), root distribution, and water use efficiency (Fang *et al.*, 2021).

### Management strategies promoting grain number are effective in enhancing yield stability

Since agronomic management can be chosen by the farmer, it is of interest to discover which management is optimal for yield stability. In general, management that promotes canopy growth also increases SN and therefore GY. Our findings demonstrated that the management superiority index of GY (*P*_m,GY_) is most stable under optimal nitrogen levels, split application, and early sowing date (Fig. 1B). As expected, increasing nitrogen availability promotes yield stability (Macholdt and Honermeier, 2019; Martre *et al.*, 2024). Interestingly, the effectiveness of achieving yield stability by nitrogen application time and sowing date differed between nitrogen levels (Fig. 1B). In general, management that promotes GN increases *P*_m,GY_ (Figs 2E, 7). Under optimal nitrogen conditions, early sowing may enhance root development (Rasmussen and Thorup-Kristensen, 2016), which allows for improved nitrogen uptake later in the season, ultimately benefiting tiller number and GN. In contrast, under suboptimal nitrogen levels, early nitrogen availability may stimulate tiller growth, leading to an increase in tiller and spike formation (Lenoir *et al.* 2023) and, consequently, GN. Therefore, improving our understanding of nitrogen uptake, physiological mechanisms for tiller and spike growth, and GN development and its relationship with GCD and phenology will be crucial to further enhance the management-driven yield stability.

## Supplementary data

The following supplementary data are available at *JXB* online.

Fig. S1. Location of the experimental field.

Fig. S2. Details of experimental design.

Fig. S3. Visualization of daily weather conditions and the time point of destructive measurements.

Fig. S4. Visualization of volumetric soil water contents in different soil layers and the distribution of precipitation over days of year.

Fig. S5. Data of grain yield, grain number, grain per spike, spike number, thousand kernel weight, grain width, and grain length.

Fig. S6. Data of stay-green traits, phenology, and phase of phenologies.

Table S1. Information for eight elite winter wheat cultivars.

Table S2. Fertilization application plan.

Table S3. Yearly sowing date treatment plan.

Table S4. Traits measured in the field experiments.

Table S5. Definition of 41 calculated traits.

Table S6. Simple statistics for 130 physiological, agronomic, and phenological traits in field experiments from 2019 to 2021.

Table S7. Ranges of environmental variables between years 2020 and 2021 from sowing to maturity.

eraf191_suppl_Supplementary_Figures_S1-S6

eraf191_suppl_Supplementary_Tables_S1-S7

## Data Availability

All data, and reproducible codes for data-analyses are available under https://github.com/Illustratien/JXB_analysis.

## Abbreviations:

DM: dry matter
GCA: green canopy area
GCD: green canopy duration
GL: grain length
GN: grain number
GpS: grain per spike
GW: grain width
GY: grain yield
LAI: leaf area index
LI: light interception
SI: stability index
SN: spike number
TGW: thousand grain weight
TT: thermal sum
WSC: water soluble carbohydrate.
