# Supplementary material for: Modern elite winter wheat cultivars use two physiological pathways to achieve yield stability
Source: J Exp Bot. 2025 Jun 7;77(9):2727–42. doi: 10.1093/jxb/eraf191 (PMC13139659; doi:10.1093/jxb/eraf191)
Supplement: eraf191_suppl_Supplementary_Figures_S1-S6 [file eraf191_suppl_supplementary_figures_s1-s6.pdf]

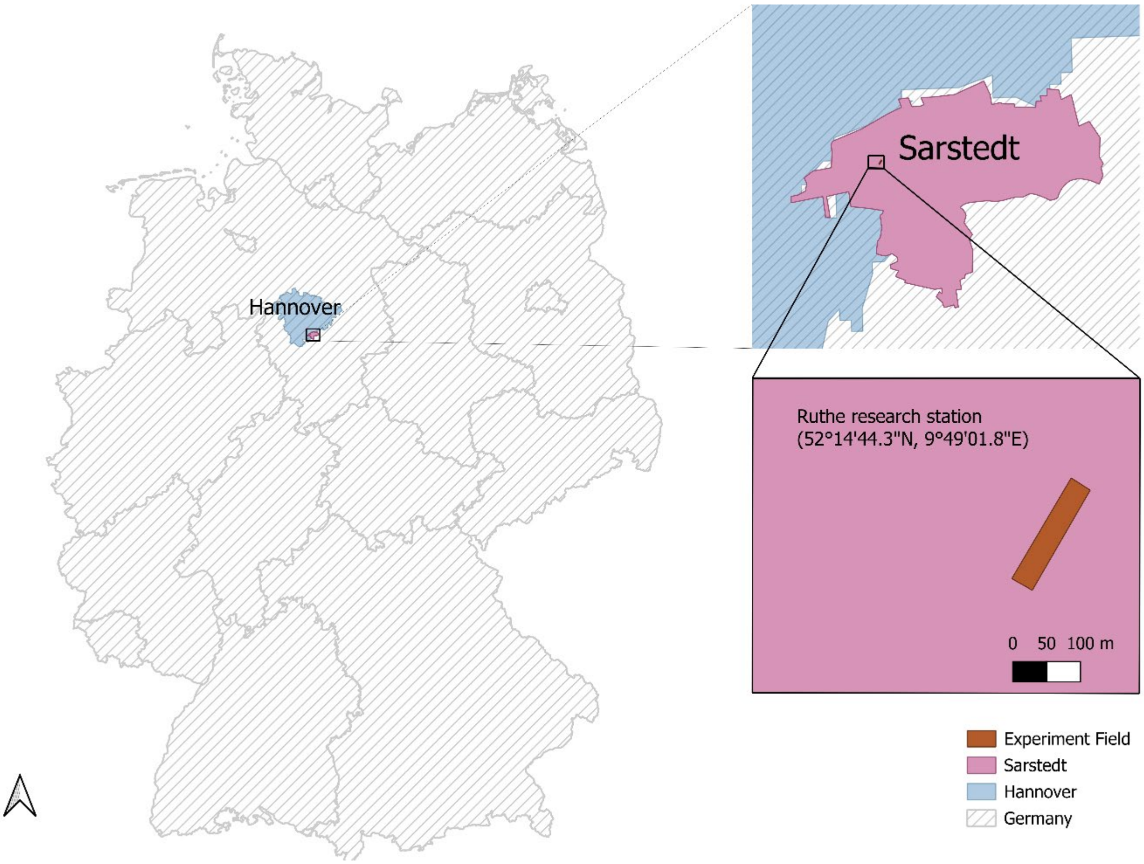

Figure S1. Location of experimental field.

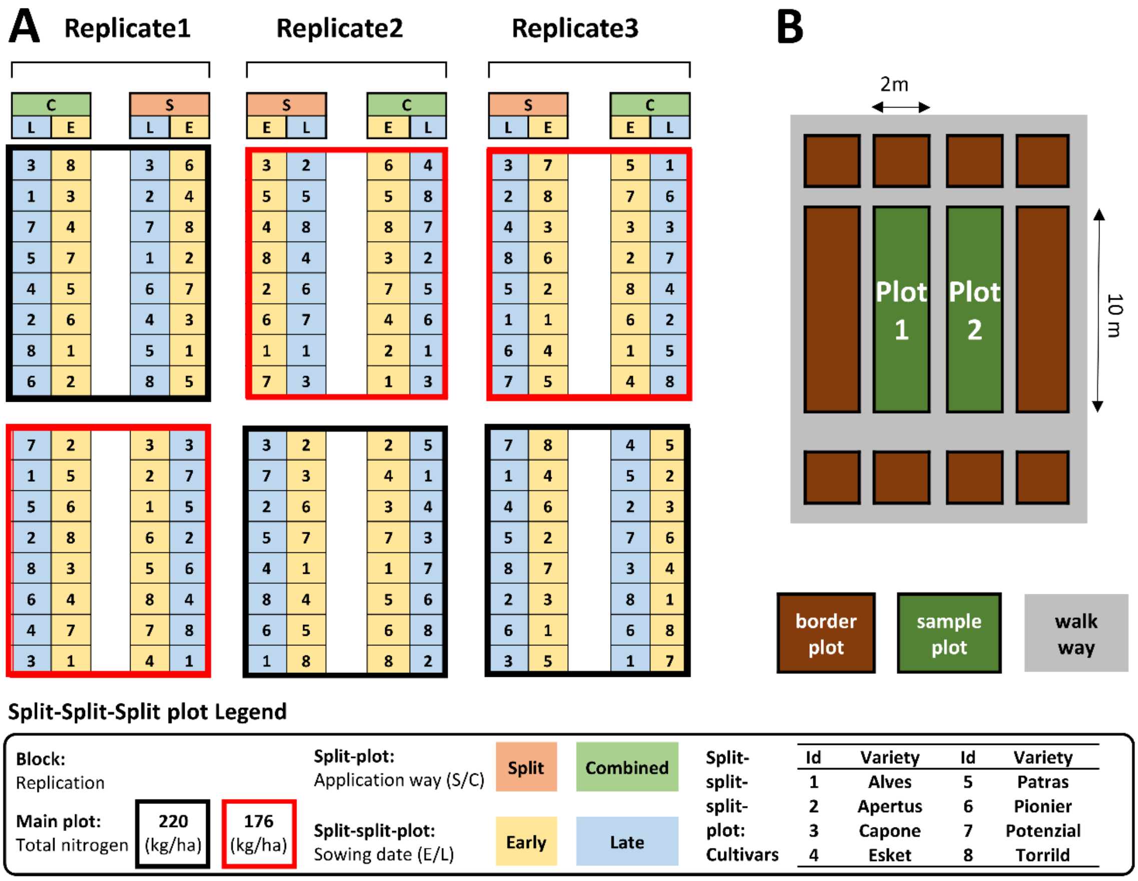

Figure S2. Detail of experimental design.

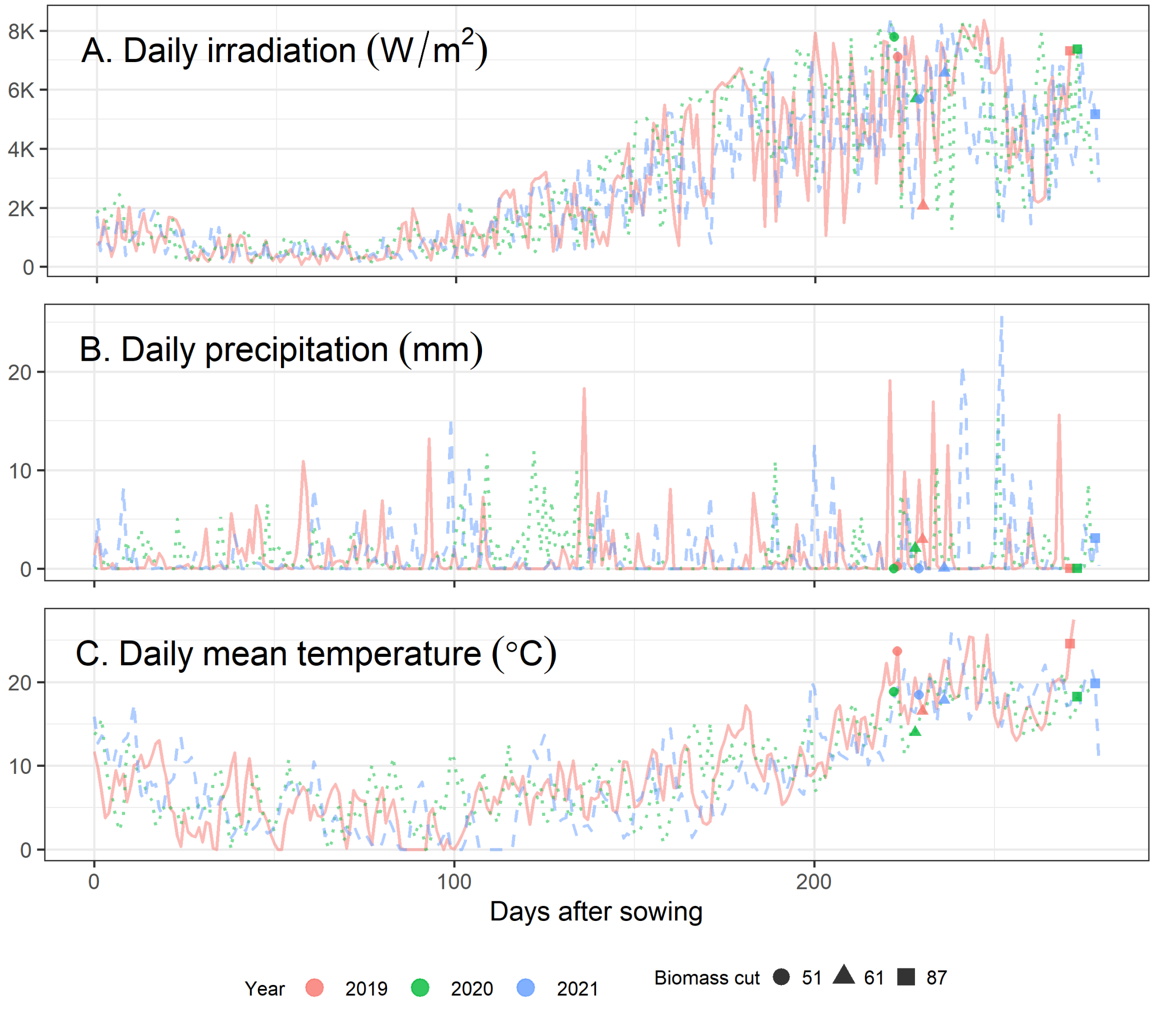

Figure S3. Visualization of daily weather conditions and the time point of destructive measurements.

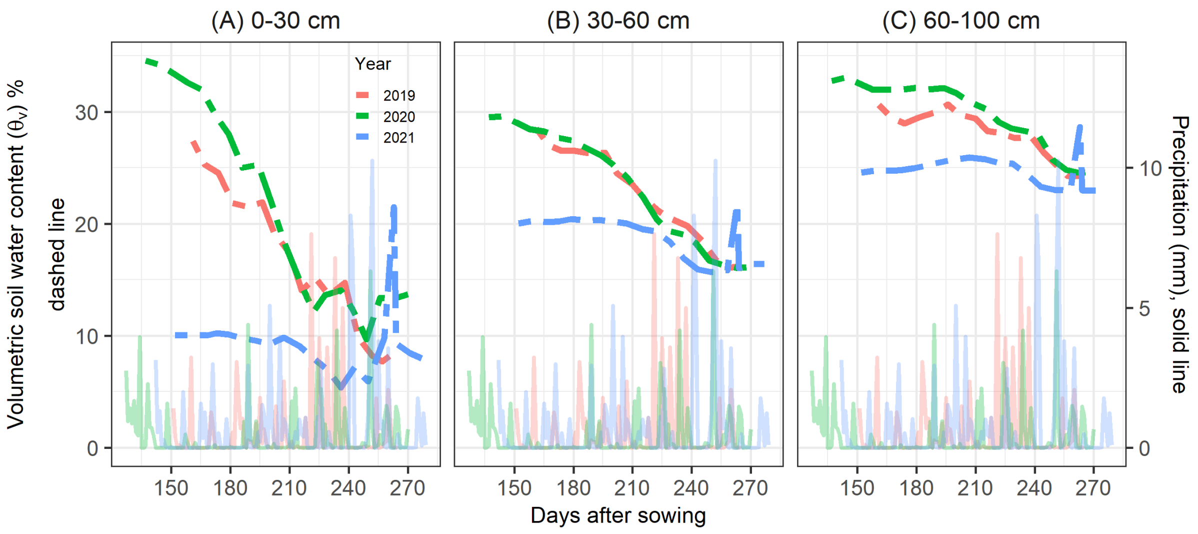

Figure S4. Visualization of volumetric soil water contents (Θv) in different soil layer and the distribution of precipitation over days of year.

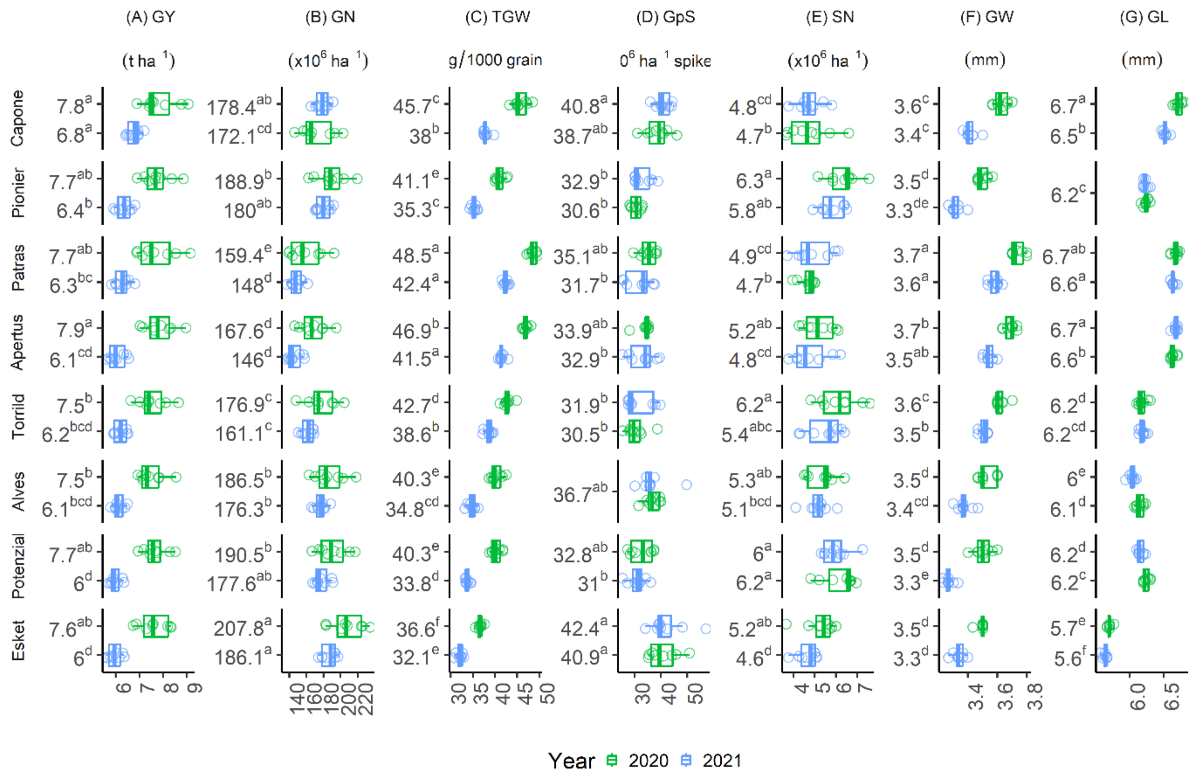

Figure S5. Data of grain yield, grain number, grain per spike, spike number, thousand kernel weight, grain width and grain length. Abbreviation: grain yield (GY), grain number (GN), grain per spike (GpS), spike number (SN), thousand grain weight (TGW), grain width (GW), grain length (GL).

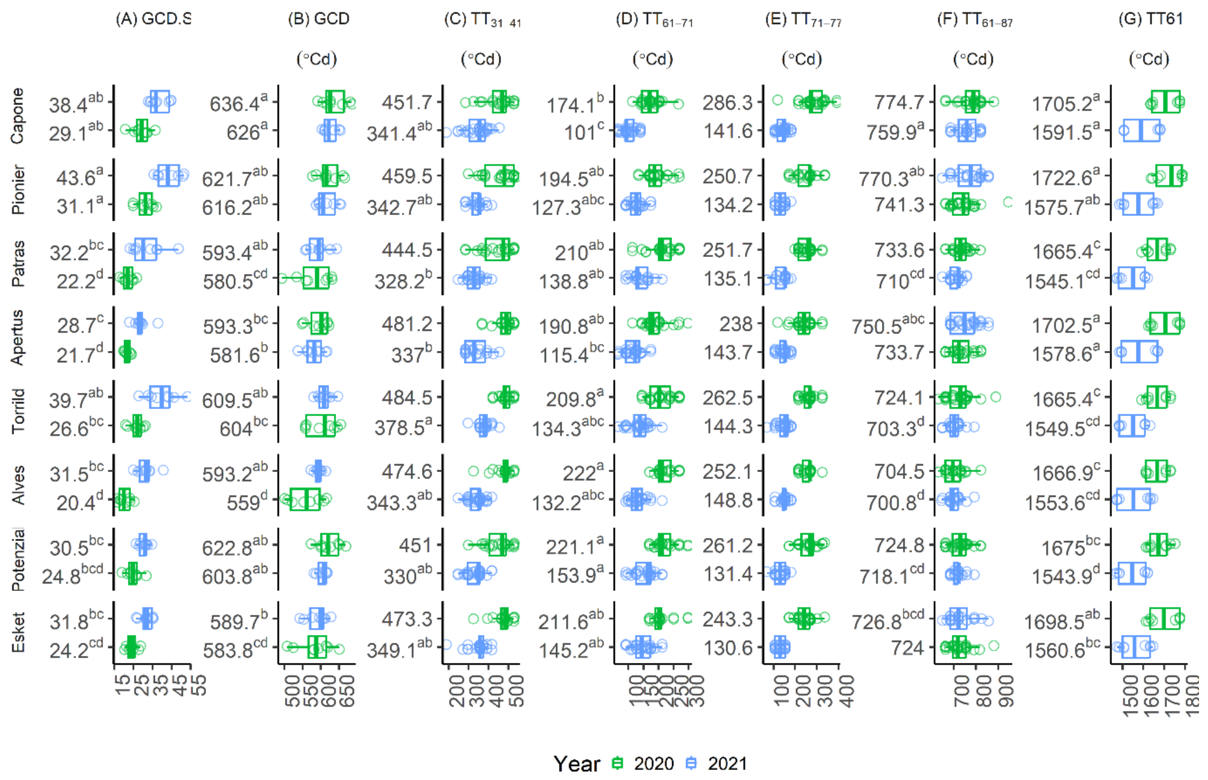

Figure S6. Data of stay-green traits, phenology and phase of phenologies. Abbreviation: slope of green canopy decade (GCD.S), green canopy duration (GCD), phenological phase between BBCH31 and BBCH41 (TT_{31-41}), phenological phase between BBCH61 and BBCH87 (TT_{61-87}), phenological phase between BBCH61 and BBCH71 (TT_{61-71}), time to flowering (TT_{61}).
